# Cigarette smoking and disproportionate changes of thoracic skeletal muscles in low-dose chest computed tomography

**DOI:** 10.1038/s41598-023-46360-0

**Published:** 2023-11-17

**Authors:** Woo Hyeon Lim, Suhyun Jeong, Chang Min Park

**Affiliations:** 1 Department of Radiology, Seoul National University Hospital, 101 Daehak-ro, Jongno-gu, Seoul, 03080 Republic of Korea; 2 Department of Radiology, Namwon Medical Center, 365 Chungjeong-no, Namwon, Jeollabuk-do 55726 Republic of Korea; 3 Department of Radiology, Seoul National University College of Medicine, 101 Daehak-ro, Jongno-gu, Seoul, 03080 Republic of Korea; 4 Institute of Radiation Medicine, Seoul National University Medical Research Center, Seoul, Republic of Korea; 5 Institute of Medical and Biological Engineering, Medical Research Center, Seoul National University, Seoul, Republic of Korea

**Keywords:** Computed tomography, Skeletal muscle

## Abstract

Association between smoking intensity and the quantity and quality of thoracic skeletal muscles (TSMs) remains unexplored. Skeletal muscle index (SMI; skeletal muscle area/height^2^) and percentage of normal attenuation muscle area (NAMA%) were measured to represent the quantity and quality of the skeletal muscles, respectively, and quantification was performed in pectoralis muscle at aortic arch (AA-PM), TSM at carina (C-TSM), erector spinae muscle at T12 (T12-ESM), and skeletal muscle at L1 (L1-SM). Among the 258 men (median age, 62 years [IQR: 58–69]), 183 were current smokers (median smoking intensity, 40 pack-years [IQR: 30–46]). SMI and NAMA% of AA-PM significantly decreased with pack-year (*β* =  − 0.028 and − 0.076; *P* < 0.001 and *P* = 0.021, respectively). Smoking intensity was inversely associated with NAMA% of C-TSM (*β* =  − 0.063; *P* = 0.001), whereas smoking intensity showed a borderline association with SMI of C-TSM (*β* =  − 0.023; *P* = 0.057). Smoking intensity was associated with the change in NAMA% of L1-SM (*β* =  − 0.040; *P* = 0.027), but was not associated with SMI of L1-SM (*P* > 0.05). Neither NAMA% nor SMI of T12-ESM was affected by smoking intensity (*P* > 0.05). In conclusion, smoking intensity was associated with the change of TSMs. Its association varied according to the location of TSMs, with the most associated parts being the upper (AA-PM) and middle TSMs (C-TSM).

## Introduction

Efforts have been made to measure skeletal muscle areas using chest computed tomography (CT) and integrate this information into patient care^1–9^. For example, the utilization of skeletal muscle areas as prognostic factors in patients with lung cancer or chronic obstructive pulmonary disease (COPD) has been explored^2, 3^. In addition, functional and physiologic statuses, such as forced vital capacity and 6-min walk distance, are indirectly inferred using thoracic skeletal muscle (TSM) areas^3–5^.

Recently, a dose-dependent relationship between smoking and the lower TSM area at the carina to the skeletal muscle area at the first lumbar vertebra ratio has been reported^10^, suggesting that smoking be associated with disproportionate wasting of the TSM. Smoking can induce skeletal muscle dysfunction by accelerating proteolysis and inhibiting protein synthesis^11^, which cannot explain how smoking is associated with disproportionate TSM wasting.

In addition, previous studies have simply focused on the quantity change of TSMs according to the smoking history^10, 12, 13^, but both the quantity and quality of the TSM should be investigated to understand smoking-associated TSM changes. Since COPD can induce skeletal muscle dysfunction by intrinsic mechanism (at the molecular or cellular levels), extrinsic mechanism (i.e. chest wall remodeling), and mixed mechanisms (i.e. interdependence of locomotor and ventilatory muscles)^14, 15^, COPD should be considered as an important mediator between smoking and disproportionate TSM wasting. 

For the analysis of patients with COPD, a commonly used method is to adjust for the results of the pulmonary function test (PFT), including forced expiratory volume in 1 s^13^. However, PFT is not a routine practice in medical check-ups or lung cancer screening and is known to be less sensitive in the early stages of COPD^16^. Conversely, morphologic changes associated with COPD, such as centrilobular emphysema, total lung capacity (TLC), or bronchial wall thickening, can be easily identified using chest CT^17, 18^.

Thus, this study aimed to investigate the association between smoking intensity and the quantity and quality of the TSM after adjusting for COPD-associated changes on low-dose chest CT. 

## Results

**Figure 1:**
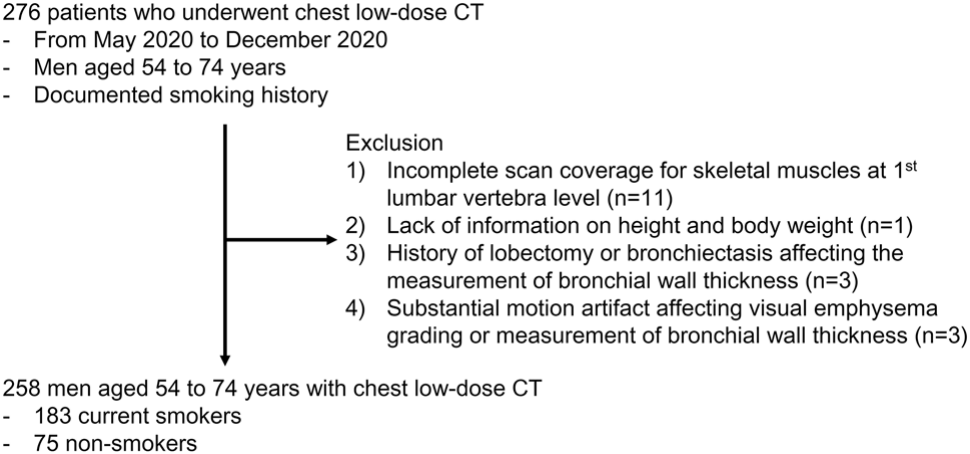
Study flow diagram.

Among the 258 men (median age, 62 years, [interquartile range (IQR): 58–69]) who were finally included, 183 were current smokers (median age, 61 years [IQR: 58–66]; median smoking intensity, 40 PY [IQR: 30–46]), whereas 75 were non-smokers (median age, 68 years [IQR: 62–71]) (Fig. 1). According to the smoking intensity (non-smoker vs. smokers with PY < 40 vs. smokers with PY ≥ 40), age, body-mass index (BMI), PY, skeletal muscle index (SMI) at the first lumbar vertebra (L1-SMI), percentage of normal attenuation skeletal muscle area (NAMA%) at the first lumbar vertebra (L1-SNAMA%), thoracic skeletal muscle index at the carina (C-TSMI), percentage of normal attenuation thoracic skeletal muscle area at the carina (C-TSNAMA%), pectoralis muscle index at the aortic arch (AA-PMI), square root of the wall area at internal perimeter 10 (SRWA-PI10), visual emphysema grade (VEG), and TLC were significantly different (*P* < 0.05), whereas percentage of normal attenuation pectoralis muscle area at the aortic arch (AA-PNAMA%), erector spinae muscle index at the 12th thoracic vertebra (T12-ESMI), and percentage of normal attenuation erector spinae muscle area at the 12th thoracic vertebra (T12-ESNAMA%) were comparable between the three groups (*P* > 0.05) (Table 1).

**Table 1:** Comparison of characteristics and CT-based morphologic changes according to smoking intensity.

Variables*	Non-smoker (n = 75)	Smokers with pack-year < 40 (n = 73)	Smokers with pack-year ≥ 40 (n = 110)	*P*-values
Age (years)	68 [62, 71]	58 [56, 62]	63.5 [60, 68]	< 0.001
Body weight (kg)	68.1 [62.0, 74.0]	69.1 [64.0, 73.5]	67.0 [60.0, 72.4]	0.225
Height (cm)	165.1 ± 6.7	167.5 ± 6.4	166.2 ± 6.6	0.081
Body-mass index (kg/m^2^)	24.4 [23.3, 26.8]	24.7 [23.1, 26.1]	23.9 [22.6. 25.6]	0.048
Pack-year	0 [0, 0]	30 [30, 30]	45 [40, 50]	< 0.001
Skeletal muscles				
L1-SMI (cm^2^/m^2^)	46.3 [42.6, 49.9]	46.3 [41.7, 49.7]	43.5 [39.6, 47.4]	0.015
L1-SNAMA% (%)	62.2 [57.0, 67.1]	64.2 [58.4, 67.3]	60.5 [56.3, 64.8]	0.016
C-TSMI (cm^2^/m^2^)	39.1 ± 5.1	38.1 ± 5.0	36.2 ± 5.3	0.001
C-TSNAMA% (%)	62.0 [58.8, 66.5]	61.8 [56.4, 66.6]	59.8 [55.9, 64.1]	0.037
AA-PMI (cm^2^/m^2^)	15.4 [13.7, 17.2]	13.8 [12.7, 15.6]	13.2 [11.5, 15.2]	< 0.001
AA-PNAMA% (%)	73.8 [65.3, 78.1]	74.5 [64.6, 79.3]	70.3 [62.3, 76.9]	0.177
T12-ESMI (cm^2^/m^2^)	14.9 ± 3.1	14.9 ± 2.3	14.2 ± 2.7	0.103
T12-ESNAMA% (%)	71.3 [67.1, 76.2]	70.8 [64.0, 75.7]	68.3 [63.0, 73.7]	0.070
SRWA-PI10	5.39 [5.26, 5.49]	5.28 [5.15, 5.38]	5.34 [5.22, 5.51]	0.003
VEG				< 0.001
None	64 (85.3%)	51 (69.9%)	52 (47.3%)	
Trace	3 (4.0%)	6 (8.2%)	20 (18.2%)	
Mild	5 (6.7%)	9 (12.3%)	17 (15.5%)	
Moderate	3 (4.0%)	7 (9.6%)	9 (8.2%)	
Severe	0 (0.0%)	0 (0.0%)	12 (10.9%)	
LAA%950	9.8 [7.2, 13.0]	11.5 [9.4, 14.6]	12.5 [8.7, 18.0]	0.002
HU15%	− 933 [− 945, − 921]	− 941 [− 950, − 932]	− 943 [− 957, − 929]	0.002
Total lung capacity (L)	4.6 ± 0.8	5.5 ± 0.9	5.5 ± 0.9	< 0.001

*Numerical variables with normal distribution were provided with mean ± standard deviation, while those not showing normal distribution were provided with median [interquartile range].

L1-SMI, skeletal muscle index at the first lumbar vertebra; L1-SNAMA%, percentage of normal attenuation skeletal muscle area at the first lumbar vertebra; C-TSMI, thoracic skeletal muscle index at the carina; C-TSNAMA%, percentage of normal attenuation thoracic skeletal muscle area at the carina; AA-PMI, pectoralis muscle index at the aortic arch; AA-PNAMA%, normal attenuation pectoralis muscle area at the aortic arch; T12-ESMI, erector spinae muscle index at the 12th thoracic vertebra; T12-ESNAMA%, percentage of normal attenuation erector spinae muscle area at the 12th thoracic vertebra; SRWA-PI10, square root of the wall area at internal perimeter 10; VEG, visual emphysema grade; LAA%950, low attenuation area less than − 950 Hounsfield unit; HU15%, Hounsfield unit at 15th percentile.

The agreement for VEG was almost perfect between the two readers (κ = 0.88). Low attenuation area less than − 950 HU (LAA%950) and HU at 15th percentile (HU15%) are plotted according to VEG in Supplementary Fig. 1.

### Smoking intensity and SM quantity

**Figure 2:**
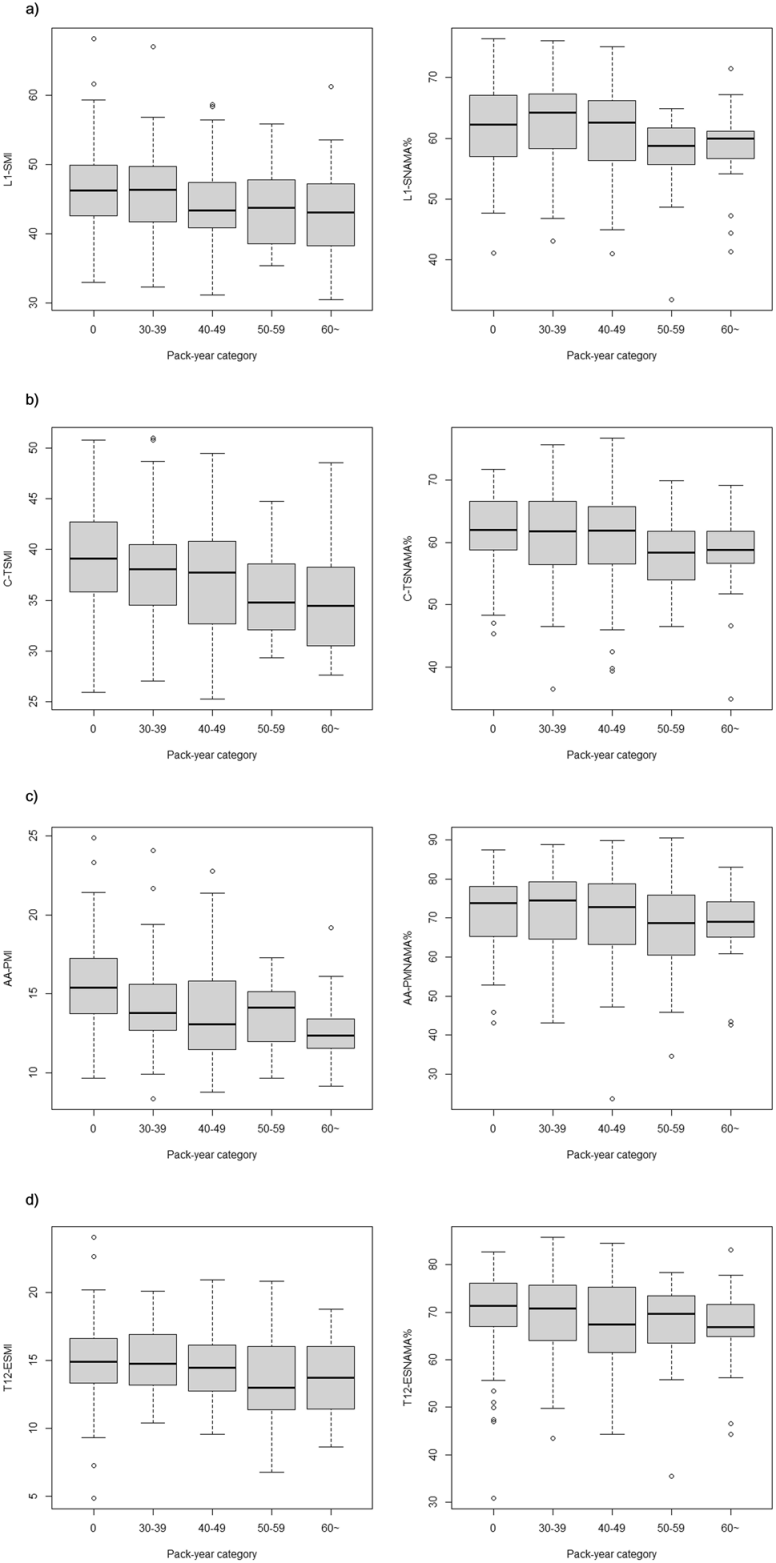
The quantity and quality of skeletal muscles according to smoking intensity: (**a**) skeletal muscle at the first lumbar vertebra level (L1-SMI, skeletal muscle index at the first lumbar vertebra; L1-SNAMA%, percentage of normal attenuation skeletal muscle area at the first lumbar vertebra), (**b**) thoracic skeletal muscle at the carina level (C-TSMI, thoracic skeletal muscle index at the carina; C-TSNAMA%, percentage of normal attenuation thoracic skeletal muscle area at the carina), (**c**) pectoralis muscle at the aortic arch level (AA-PMI, pectoralis muscle index at the aortic arch; AA-PNAMA%, normal attenuation pectoralis muscle area at the aortic arch), and (**d**) erector spinae muscle at the 12th thoracic vertebra level (T12-ESMI, erector spinae muscle index at the 12th thoracic vertebra; T12-ESNAMA%, percentage of normal attenuation erector spinae muscle area at the 12th thoracic vertebra).

The distribution of SMI for every 10 PY is presented in Fig. 2. The SMI tended to decrease with increasing PY.

Smoking intensity was inversely associated with AA-PMI in all the models (*β*, − 0.034 to − 0.027; *P* < 0.001); the coefficient of PY in Model 7 was − 0.028 (95% confidence interval [CI]: − 0.044, − 0.013; *P* < 0.001). Similarly, smoking intensity showed a significant or borderline association with C-TSMI (*β*, − 0.036 to − 0.023; *P* < 0.001 to 0.057); the association between smoking intensity and C-TSMI tended to be attenuated more significantly when VEG was included in the model (Model 7, *β* = -0.023; 95% CI: − 0.047, 0.001; *P* = 0.057). In contrast, smoking intensity was not associated with T12-ESMI or L1-SMI (*P* > 0.05) (Table 2, Supplementary Table 1).

**Table 2:** Effect of pack-year of smoking on skeletal muscle quantity.

Model	C-TSMI	*P*-values	AA-PMI	*P*-values	T12-ESMI	*P*-value	L1-SMI*	*P*-value
Model 1	− 0.028 (− 0.051, − 0.006)	0.013	− 0.031 (− 0.046, − 0.017)	< 0.001	0.000 (− 0.011, 0.011)	0.981	− 0.018 (− 0.048, 0.012)	0.239
Model 2	− 0.030 (− 0.052, − 0.007)	0.009	− 0.028 (− 0.043, − 0.014)	< 0.001	− 0.003 (− 0.014, 0.008)	0.625	− 0.024 (− 0.054, 0.006)	0.110
Model 3	− 0.036 (− 0.057, − 0.015)	< 0.001	− 0.034 (− 0.047, − 0.020)	< 0.001	− 0.003 (− 0.013, 0.008)	0.633	− 0.022 (− 0.050, 0.006)	0.123
Model 4	− 0.023 (− 0.047, 0.000)	0.050	− 0.027 (− 0.042, − 0.011)	< 0.001	− 0.001 (− 0.012, 0.011)	0.924	− 0.020 (− 0.051, 0.012)	0.215
Model 5	− 0.028 (− 0.051, − 0.006)	0.013	− 0.032 (− 0.047, − 0.017)	< 0.001	0.000 (− 0.011, 0.011)	0.963	− 0.018 (− 0.048, 0.012)	0.236
Model 6	− 0.029 (− 0.052, − 0.007)	0.011	− 0.030 (− 0.044, − 0.015)	< 0.001	− 0.004 (− 0.015, 0.007)	0.439	− 0.025 (− 0.056, 0.005)	0.098
Model 7	− 0.023 (− 0.047, 0.001)	0.057	− 0.028 (− 0.044, − 0.013)	< 0.001	− 0.002 (− 0.014, 0.009)	0.693	− 0.021 (− 0.053, 0.011)	0.197

All models were adjusted for age, body-mass index, skeletal muscle index at the first lumbar vertebra, and chronic obstructive pulmonary disease associated features: Model 1, visual emphysema grade (VEG) only; Model 2, total lung capacity (TLC) only; Model 3, square root of the wall area at internal perimeter of 10 mm (SRWA-PI10) only; Model 4, VEG and TLC; Model 5, VEG and SRWA-PI10; Model 6, TLC and SRWA-PI10; Model 7, VEG, TLC, and SRWA-PI10.

Presented values were coefficients of pack-year with 95th percentile confidence interval in parenthesis.

*All models were adjusted for age, body-mass index, and chronic obstructive pulmonary disease associated features**.**

C-TSMI, thoracic skeletal muscle index at the carina; T12-ESMI, erector spinae muscle index at the 12th thoracic vertebra; AA-PMI, pectoralis muscle index at the aortic arch; L1-SMI, skeletal muscle index at the first lumbar vertebra.

**Figure 3:**
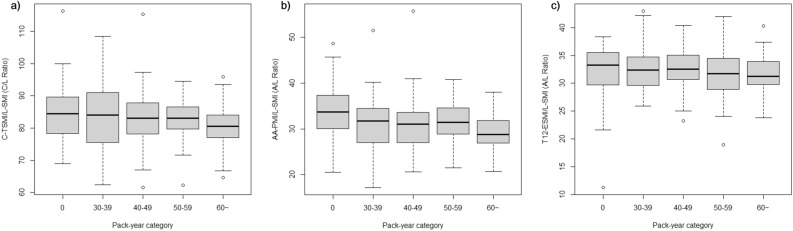
Disproportionate skeletal muscles wasting according to smoking intensity: (**a**) C/L ratio (thoracic skeletal muscle index at the carina to skeletal muscle index at the first lumbar [L1-SMI] ratio), (**b**) A/L ratio (pectoralis muscle index at the aortic arch to L1-SMI ratio), and (**c**) T/L ratio (erector spinae muscle index at the 12th thoracic vertebra to L1-SMI ratio).

As a result, smoking intensity was also associated with disproportionate TSM (at the carina level) and pectoralis muscle (at the aortic arch level) wasting: C-TSMI to L1-SMI ratio (Model 7, *β* = -0.054; 95% CI: − 0.107, − 0.002; *P* = 0.042) and AA-PMI to L1-SMI ratio (Model 7, *β* = -0.061; 95% CI: − 0.094, − 0.029; *P* < 0.001) significantly decreased with PY. However, the T12-ESMI to L1-SMI ratio was not related to smoking intensity (*P* = 0.535) (Fig. 3, Supplementary Table 2).

Smoking intensity was as important a factor as COPD-associated CT findings for the quantity of the TSM at the carina (variable importance score: PY, 1.91; severe centrilobular emphysema, 1.95; TLC, 1.32; and age, 3.15) and pectoralis muscle at the aortic arch (PY, 3.59; severe centrilobular emphysema, 1.07; TLC, 1.41; and age, 0.37) (Supplementary Fig. 2).

### Smoking intensity and SM quality

The distribution of NAMA% for every 10 PY is presented in Fig. 2. The NAMA% tended to decrease with increasing PY.

Smoking intensity was associated with C-TSNAMA% and AA-PNAMA% in all models (*β*, − 0.078 to − 0.063 and − 0.079 to − 0.074; *P* < 0.001 to 0.001 and *P* = 0.008 to 0.021, respectively). For C-TSNAMA% and AA-PNAMA%, coefficients of PY in Model 7 were − 0.063 (95% CI: − 0.101, − 0.025; *P* = 0.001) and − 0.076 (95% CI: − 0.141, − 0.011; *P* = 0.021), respectively. In addition, smoking intensity was an associated factor for L1-SNAMA% (Model 7, *β* = -0.040; 95% CI: − 0.076, − 0.005; *P* = 0.027). However, smoking intensity was not associated with T12-ESNAMA% (Model 7, *P* = 0.643) (Table 3, Supplementary Table 3).

**Table 3:** Effect of pack-year of smoking on skeletal muscle quality.

Model	C-TSNAMA%	*P*-value	AA-PNAMA%	*P*-value	T12-ESNAMA%	*P*-value	L1-SNAMA%	*P*-value
Model 1	− 0.073 (− 0.108, − 0.037)^a^	< 0.001	− 0.074 (− 0.135, − 0.014)	0.016	− 0.033 (− 0.082, 0.016)	0.188	− 0.041 (− 0.075, − 0.008)	0.015
Model 2	− 0.065 (− 0.100, − 0.029)	< 0.001	− 0.079 (− 0.140, − 0.018)	0.011	− 0.027 (− 0.076, 0.023)	0.296	− 0.045 (− 0.079, − 0.012)	0.009
Model 3	− 0.078 (− 0.112, − 0.044)	< 0.001	− 0.078 (− 0.136, − 0.021)	0.008	− 0.051 (− 0.099, − 0.004)	0.034	− 0.051 (− 0.083, − 0.020)	0.002
Model 4	− 0.063 (− 0.100, − 0.025)	0.001	− 0.077 (− 0.141, − 0.013)	0.018	− 0.012 (− 0.063, 0.039)	0.640	− 0.038 (− 0.073, − 0.003)	0.034
Model 5	− 0.073 (− 0.109, − 0.038)	< 0.001	− 0.074 (− 0.135, − 0.013)	0.017	− 0.034 (− 0.083, 0.015)	0.177	− 0.042 (− 0.075, − 0.009)	0.014
Model 6	− 0.065 (− 0.101, − 0.029)	< 0.001	− 0.078 (− 0.139, − 0.016)	0.014	− 0.027 (− 0.077, 0.024)	0.300	− 0.047 (− 0.081, − 0.013)	0.007
Model 7	− 0.063 (− 0.101, − 0.025)	0.001	− 0.076 (− 0.141, − 0.011)	0.021	− 0.012 (− 0.064, 0.040)	0.643	− 0.040 (− 0.076, − 0.005)	0.027

All models were adjusted for age, body-mass index, skeletal muscle index at the first lumbar vertebra, and chronic obstructive pulmonary disease associated features: Model 1, visual emphysema grade (VEG) only; Model 2, total lung capacity (TLC) only; Model 3, square root of the wall area at internal perimeter of 10 mm (SRWA-PI10) only; Model 4, VEG and TLC; Model 5, VEG and SRWA-PI10; Model 6, TLC and SRWA-PI10; Model 7, VEG, TLC, and SRWA-PI10.

Presented values were coefficients of pack-year with 95th percentile confidence interval in parenthesis.

C-TSNAMA%, percentage of normal attenuation thoracic skeletal muscle area at the carina; AA-PNAMA%, normal attenuation pectoralis muscle area at the aortic arch; T12-ESNAMA%, percentage of normal attenuation erector spinae muscle area at the 12th thoracic vertebra; L1-SNAMA%, percentage of normal attenuation skeletal muscle area at the first lumbar vertebra.

Smoking intensity was more important than COPD-associated CT findings for C-TSNAMA% (variable importance score: PY, 3.30; severe centrilobular emphysema, 2.74; TLC, 1.57; and age, 4.87) and AA-PNAMA% (PY, 2.32; severe centrilobular emphysema, 1.32; TLC, 0.19; and age, 1.49) (Supplementary Fig. 2).

**Figure 4:**
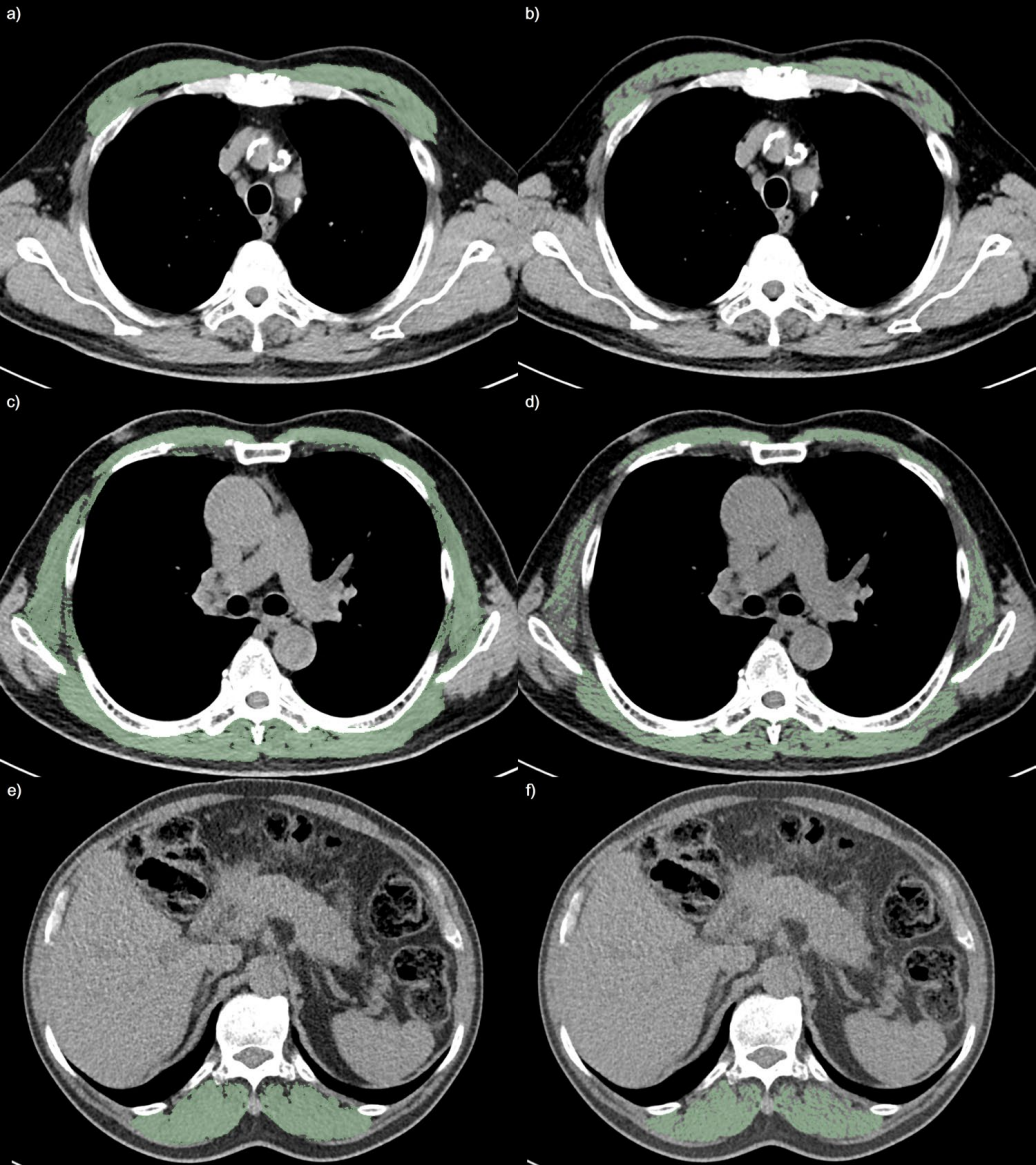
Thoracic skeletal muscle change in a current smoker: In a 68 year-old current smoker with 40 pack-year (body weight 58.2 kg, height 157.4 cm, body-mass index 23.5 kg/m^2^) showed severe degree of centrilobular emphysema, total lung capacity of 4.3L, and 5.22 mm of the square root of the wall area at internal perimeter of 10 mm: (**a**,**b**) pectoralis muscle index at the aortic arch (AA-PMI) and percentage of normal attenuation pectoralis muscle area at the aortic arch (AA-PNAMA%) were 10.7cm^2^/m^2^ and 61.4%, respectively; (**c**,**d**) thoracic skeletal muscle index at the carina (C-TSMI) and percentage of normal attenuation thoracic skeletal muscle area at the carina (C-TSNAMA%) were 34.4cm^2^/m^2^ and 47.6%, respectively; and (**e**,**f**) erector spinae muscle index at the 12th thoracic vertebra (T12-ESMI) and percentage of normal attenuation erector spinae muscle area at the 12th thoracic vertebra (T12-ESNAMA%) were 13.9cm^2^/m^2^ and 65.8%, respectively. The SMI at the first lumbar vertebra (L1-SMI) and percentage of normal attenuation skeletal muscle area at the first lumbar vertebra (L1-SNAMA%) were 42.1cm^2^/m^2^ and 53.5%, respectively (not presented).

**Figure 5:**
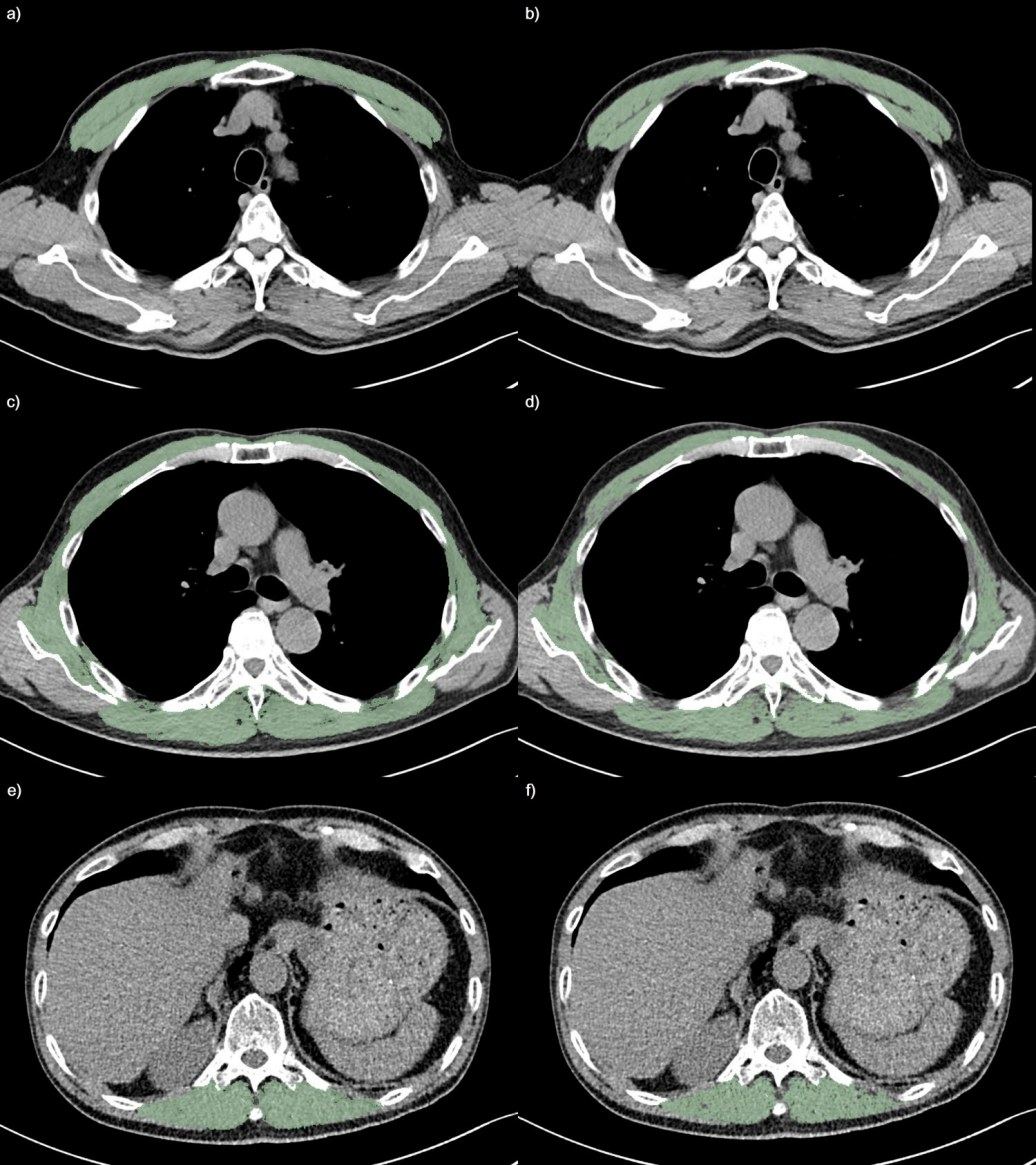
Thoracic skeletal muscle change in a non-smoker: In a 69 year-old men without documented smoking history (body weight 71 kg, height 172.7 cm, body-mass index 23.8 kg/m^2^) showed absence of centrilobular emphysema, total lung capacity of 5.2L, and 5.06 mm of square root of the wall area at internal perimeter of 10 mm: (**a**,**b**) pectoralis muscle index at the aortic arch (AA-PMI) and percentage of normal attenuation pectoralis muscle area at the aortic arch (AA-PNAMA%) were 13.9cm^2^/m^2^ and 77.8%, respectively; (**c**,**d**) thoracic skeletal muscle index at the carina (C-TSMI) and percentage of normal attenuation thoracic skeletal muscle area at the carina (C-TSNAMA%) were 36.4cm^2^/m^2^ and 64.7%, respectively; and (**e**,**f**) erector spinae muscle index at the 12th thoracic vertebra (T12-ESMI) and percentage of normal attenuation erector spinae muscle area at the 12th thoracic vertebra (T12-ESNAMA%) were 12.2cm^2^/m^2^ and 77.6%, respectively. The SMI at the first lumbar vertebra (L1-SMI) and percentage of normal attenuation skeletal muscle area at the first lumbar vertebra (L1-SNAMA%) were 40.6cm^2^/m^2^ and 68.4%, respectively (not presented).

Representative cases of TSM changes in smokers and non-smokers are presented in Figs. 4 and 5, respectively.

## Discussion

This study demonstrated that the relationship between smoking intensity and the changes of skeletal muscle on low-dose chest CT varied by anatomic locations, and the quantity and quality of TSMs were generally associated with smoking intensity. However, the association between change of erector spinae muscle at the 12th thoracic vertebra level and smoking intensity was not evident. In addition, the quality of skeletal muscle at the first lumbar vertebra level as defined by CT was inversely associated with PY, but the quantity did not change with smoking intensity.

Diaz et al.^13^ reported that the pectoralis muscle area and index at the aortic arch level were independent prognostic factors in smokers without airflow limitation from COPDGene cohorts, whereas the erector spinae muscle was not prognostic. In a previous study^12^, baseline lean body mass was comparable between individuals with airflow limitation and current smokers without evidence of airflow limitation in men aged 70–79 years. In these studies^12, 13^, the relationship between smoking intensity and TSM changes was not comprehensively investigated. Lim et al.^10^ reported a dose-dependent disproportionate decrease in TSM at the carina level according to PY; however, other parts of the TSMs were not investigated, and COPD-associated CT findings were not considered, which might be the most important mediator between smoking and TSM wasting.

This study investigated the relationship between smoking intensity and TSM changes in various body parts in terms of muscle quantity and quality, after adjusting for several important confounders or mediators, such as age, BMI, and COPD-associated CT findings. Interestingly, the association between the smoking intensity and TSMs seemed to vary according to the anatomical location of the TSMs, which may be related to their role in respiration. Because the external intercostal, pectoralis, trapezius, and serratus anterior muscles are the assessed parts of the TSMs at the carina level, TSMs at this level might serve as inspiratory accessory muscles^19, 20^. Conversely, skeletal muscles at the first lumbar vertebra level partly contribute to either respiration (i.e., accessory expiratory muscles: anterolateral abdominal wall muscles) or work against gravitation (i.e., antigravity muscles: erector spinae muscles)^3, 21^. Our results might suggest that the greater the degree of involvement in respiration, the greater the effect of smoking intensity on TSM quantification. These results might also suggest that the subclinical work of breathing exist in heavy smokers, which is not fully captured on the PFT^16^.

On the basis of these results, the use of skeletal muscles at the first lumbar vertebra level or erector spinae muscles at the 12th thoracic vertebra level may be recommended for the quantification of skeletal muscles on low-dose chest CT, as these parts are less significantly associated with smoking. In addition, smoking intensity should always be considered when assessing the TSM at the carina and pectoralis muscle at the aortic arch levels^1, 3–5, 7, 22, 23^, because smoking intensity is as important as COPD-associated CT findings in the quantification of these TSMs.

The association between the smoking intensity and TSM change was attenuated when VEG was included in the model, whereas the attenuation was less significant after adjusting for bronchial wall thickness. These findings were consistent with those of previous studies that showed that skeletal muscle wasting was more common in emphysema-predominant COPD than in chronic bronchitis^4, 22^. Since the structural pattern of the airway is substantially different between sexes^24^, these results need to be validated in women.

This study had some limitations. As this was a retrospective study, an unidentified bias could exist. For example, PY were a self-reported measure that was vulnerable to variability. Because PY based on medical records tend to be under-reported^25^, the association between smoking and TSM changes might be accentuated. In addition, the influence of underlying diseases could not be identified in this study population because this information was absent in some individuals. However, the presence of hypertension, diabetes, and dyslipidemia did not show a significant effect on sarcopenia in a previous study^10^. Thus, a focused evaluation of the association between TSM changes, smoking, and COPD-associated CT findings was considered acceptable. Recently, history of severe acute respiratory syndrome coronavirus-2 disease (COVID-19) has emerged as a novel cause of skeletal muscle changes^26^. As the prevalence of severe COVID-19 was extremely low in our country during the study period^27^, it could be assumed that the impact of COVID-19 on the results of this study seemed to be minimal. As current female smokers are rare and the skeletal muscles of women are significantly smaller than those of men, this study only included men. Although appropriate validation is required in women, we expect that differences due to sex may not be significant. While this study provided a consistent association between smoking intensity and disproportionate TSM wasting in a temporally different dataset from that of a previous study^10^, the clinical importance of disproportionate TSM wasting needs to be explored in future studies. It is also needed to evaluate whether disproportionate TSM wasting is observed in heavy smokers aged over 74 years. As systemic inflammation could also contribute to the skeletal muscle wasting in smokers, it might be important to assess the level of systemic inflammatory markers^11^. Finally, although a substantial number of current smokers could exhibit COPD-associated CT abnormalities without airflow limitation^28^, COPD-associated CT findings were adjusted as a possible mediator between smoking and TSM wasting rather than PFT results. Thus, evaluation after adjusting for PFT results and systemic inflammatory markers might provide comprehensive understand for the relationship between smoking, airflow limitation, systemic inflammation and TSM changes^11, 29^, and our results need to be validated under such study design.

In conclusion, the association between smoking intensity and TSM changes in terms of quantity and quality varied according to the location of the TSMs. Thus, quantification of skeletal muscles at the lower thoracic or upper lumbar level may be recommended to explore the clinical importance in patient care using chest CT. Smoking intensity should be considered when the upper or middle TSMs are used for quantification. The clinical importance of disproportionate TSM wasting in heavy smokers should be explored in future studies.

## Materials and methods

This retrospective study was approved by Public Institutional Review Board designated by Ministry of Health and Welfare, Republic of Korea (approval number: P01-202102-21-011) with a waiver of informed consent. All studies were performed in accordance with relevant guidelines and regulations.

### Patients

Between May and December 2020, consecutive patients who underwent low-dose chest CT without intravenous contrast medium administration at the Namwon Medical Center were retrospectively recruited for this study. These patients comprised those who underwent low-dose chest CT as part of a national lung cancer screening program in the Republic of Korea^30^ or their medical check-ups^31, 32^.

The inclusion criteria were as follows: 1) men aged 54–74 years and 2) a documented history of smoking. Patients were excluded from this study for the following reasons: 1) incomplete scan coverage of skeletal muscles at the first lumbar vertebra (n = 11); 2) lack of information on height and body weight (n = 1); 3) history of lobectomy or bronchiectasis that could affect bronchial wall thickness measurement (n = 3); and 4) substantial motion artifacts affecting VEG or bronchial wall thickness measurement (n = 3; Fig. 1).

### CT imaging protocols

CT imaging was performed using SOMATOM Definition AS + (Siemens) at 50 mAs/100 kVp. The CT images were reconstructed using three methods: 1) axial images with a 1.0-mm slice thickness and I50f. filter, 2) axial images with a 2.5-mm slice thickness and I30f. filter, and 3) coronal images with a 2.5-mm slice thickness and I50f. filter.

### SM segmentation

With axial images of 2.5-mm thickness, the SMAs were segmented semi-automatically using an open-source software program (3D Slicer Chest Imaging Platform, version 4.10.2) with fine manual editing by one of the authors (W.H.L.; 8 years of experience in chest imaging). HU ranging from − 29 to 150 were considered skeletal muscles, and HU ranging from 30 to 150 were classified as a normal attenuation skeletal muscle area^33^. For this study, skeletal muscle area at the first lumbar vertebra^1, 6, 7^, erector spinae muscle area at the 12th thoracic vertebra level^3, 8, 9^, thoracic skeletal muscle area at the carina level^5^, and pectoralis muscle area at the aortic arch level^1, 3, 4, 7^ were segmented. The AA-PMI to L1-SMI ratio, C-TSMI to L1-SMI ratio, and T12-ESMI to L1-SMI ratio were calculated^10^. The NAMA% (normal attenuation skeletal muscle area/total skeletal muscle area × 100) for each muscle area was calculated^34^. The SMI (skeletal muscle area/height^2^) and NAMA% were representative of the quantity and quality of each skeletal muscle, respectively. The L1-SMI was representative of lean body mass on chest CT^10^. The pectoralis muscle at the aortic arch, TSM at the carina, and erector spinae muscle at the 12th thoracic vertebra level were representatives of the upper, middle, and lower TSMs, respectively.

### COPD-associated changes

To evaluate the bronchial wall thickness, SRWA-PI10 was calculated as previously described by the author (W.H.L.)^35, 36^, with 1.0-mm–thick images using the zero-crossing method (3D Slicer). Small airways (internal perimeter < 6 mm) and airways with bronchiectasis were carefully avoided^35^. The TLC was obtained with 1.0-mm–thick images using 3D Slicer, and the TLC was not adjusted for the height^37^.

To evaluate the degree of centrilobular emphysema, VEG (absent, trace, mild, moderate, or severe) was independently performed by two radiologists (W.H.L. and S.J.; 30 years of experience in chest imaging)^17^. Patients’ information on smoking PY was blinded during VEG. Confluent and advanced destructive emphysema was classified as severe^17^. The presence of paraseptal emphysema was not considered in this study, as the standardized visual grading system for paraseptal emphysema is lacking and the attribution of paraseptal emphysema to extrapulmonary and systemic abnormalities is less significant than that of centrilobular emphysema^26^. The discrepancies between the two readers were resolved by consensus. As supplementary data, LAA%950 and HU15% were also calculated using 3D Slicer^38, 39^.

### Clinical information

Anthropometric data (height and body weight) and smoking status were collected from electronic medical records. The BMI (body weight/height^2^) was calculated using anthropometric data. The PY data collected from current smokers. Underlying medical diseases were not included in this study because such information is not routinely collected in the national lung cancer screening program.

### Statistical analyses

Comparisons of numerical variables were performed using a one-way analysis of variance or the Kruskal–Wallis test, as appropriate, after evaluating the normality of the parameters. Frequencies were compared using the chi-square test.

Agreement of VEG between the two readers was evaluated using Cohen’s Kappa coefficient (κ), and the κ was interpreted as follows: 1) slight agreement (κ, 0.01–0.20), 2) fair agreement (κ, 0.21–0.40), 3) moderate agreement (κ, 0.41–0.60), 4) substantial agreement (κ, 0.61–0.80), and 5) almost perfect agreement (κ, 0.81–0.99)^40^.

The effects of smoking on the TSM changes were assessed after adjusting for age, BMI, L1-SMI, and COPD-associated CT changes. Seven models with combinations of VEG, TLC, and SRWA-PI10 were built to assess the effects of smoking (Model 1: VEG only; Model 2: TLC only; Model 3: SRWA-PI10 only; Model 4: VEG and TLC; Model 5: VEG and SRWA-PI10; Model 6: TLC and SRWA-PI10; and Model 7 [full model]: VEG, TLC, and SRWA-PI10). Variable importance scores were obtained for Model 7.

Statistical analyses were performed using the R (version 4.1.2). In this study, *P*-values less than 0.05 were considered statistically significant. A variance inflation factor greater than 10 indicated the presence of multicollinearity.

### Supplementary Information

Supplementary Information.

## Data Availability

The datasets generated or analyzed during the study are available from the corresponding author on reasonable request.
