# Supplementary material for: Cigarette smoking and disproportionate changes of thoracic skeletal muscles in low-dose chest computed tomography
Source: Sci Rep. 2023 Nov 17;13:20110. doi: 10.1038/s41598-023-46360-0 (PMC10656498; doi:10.1038/s41598-023-46360-0)
Supplement: Supplementary file 1 — Supplementary Information. [file 41598_2023_46360_MOESM1_ESM.docx]

# Cigarette smoking and disproportionate changes of thoracic skeletal muscles in chest low-dose computed tomography

Woo Hyeon Lim, Suhyun Jeong, Chang Min Park

**Supplementary Tables**

**Supplementary Table 1.** Detailed results of multiple linear regression analysis for skeletal muscle quantity in the full model.

| **Variables** | **C-TSMI** | ***P*-value** | **AA-PMI** | ***P*-value** | **T12-ESMI** | ***P*-value** | **L1-SMI** | ***P*-value** |
| --- | --- | --- | --- | --- | --- | --- | --- | --- |
| **Pack-year** | -0.023  (-0.047, 0.001) | 0.057 | -0.028  (-0.044, -0.013) | <0.001 | -0.002  (-0.014, 0.009) | 0.693 | -0.021  (-0.053, 0.011) | 0.197 |
| **Age** | -0.126  (-0.205, -0.047) | 0.002 | -0.010  (-0.061, 0.042) | 0.714 | -0.026  (-0.065, 0.013) | 0.194 | -0.050  (-0.156, 0.056) | 0.351 |
| **BMI** | 0.060  (-0.127, 0.248) | 0.526 | 0.142  (0.020, 0.265) | 0.023 | -0.140  (-0.233, -0.048) | 0.003 | 1.232  (1.032, 1.432) | <0.001 |
| **L1-SMI** | 0.530  (0.437, 0.623) | <0.001 | 0.139  (0.079, 0.200) | <0.001 | 0.368  (0.322, 0.414) | <0.001 | N/A | N/A |
| **VEG (trace)** | -0.068  (-1.572, 1.436) | 0.929 | -0.307  (-1.286, 0.673) | 0.538 | -0.016  (-0.759, 0.727) | 0.967 | -1.086  (-3.102, 0.931) | 0.290 |
| **VEG (mild)** | -0.051  (-1.977, 0.949) | 0.490 | 0.178  (-0.775, 1.131) | 0.713 | -0.188  (-0.911, 0.535) | 0.628 | -1.415  (-3.373, 0.544) | 0.156 |
| **VEG (moderate)** | -1.520  (-3.335, 0.296) | 0.100 | 0.045  (-1.138, 1.227) | 0.941 | -0.460  (-1.357, 0.437) | 0.314 | -0.808  (-3.245, 1.629) | 0.514 |
| **VEG (severe)** | -2.228  (-4.475, 0.019) | 0.052 | -0.793  (-2.257, 0.671) | 0.287 | -0.704  (-1.814, 0.406) | 0.213 | 1.930  (-1.080, 4.940) | 0.208 |
| **TLC** | -0.364  (-0.907, 0.178) | 0.187 | -0.253  (-0.607, 0.100) | 0.160 | 0.142  (-0.127, 0.410) | 0.299 | 0.194  (-0.535, 0.923) | 0.601 |
| **SRWA-PI10** | -0.256  (-2.613, 2.100) | 0.831 | 1.085  (-0.450, 2.620) | 0.165 | 1.181  (0.016, 2.345) | 0.047 | 0.704  (-2.462, 3.869) | 0.662 |

Note: Presented values were coefficients of pack-year with 95th percentile confidence interval in parenthesis.

Abbreviations: C-TSMI, thoracic skeletal muscle index at the carina; T12-ESMI, erector spinae muscle index at the 12^th^ thoracic vertebra; AA-PMI, pectoralis muscle index at the aortic arch; L1-SMI, skeletal muscle index at the first lumbar vertebra; BMI, body-mass index; VEG, visual emphysema grade; TLC, total lung capacity; SRWA-PI10, square root of the wall area at internal perimeter of 10 mm; N/A, not applicable.

**Supplementary Table 2.** Effect of pack-year of smoking on disproportional thoracic skeletal muscle wasting.

| **Model** | **C/L ratio** | ***P*-value** | **A/L ratio** | ***P*-value** | **T/L ratio** | ***P*-value** |
| --- | --- | --- | --- | --- | --- | --- |
| **Model 1** | -0.066 (-0.115, -0.016) | 0.009 | -0.069 (-0.100, -0.038) | <0.001 | -0.001 (-0.026, 0.024) | 0.923 |
| **Model 2** | -0.069 (-0.118, -0.020) | 0.006 | -0.061 (-0.092, -0.031) | <0.001 | -0.008 (-0.033, 0.017) | 0.528 |
| **Model 3** | -0.083 (-0.130, -0.037) | <0.001 | -0.074 (-0.103, -0.045) | <0.001 | -0.007 (-0.030, 0.016) | 0.552 |
| **Model 4** | -0.054 (-0.106, -0.003) | 0.040 | -0.057 (-0.090, -0.025) | <0.001 | -0.003 (-0.029, 0.023) | 0.802 |
| **Model 5** | -0.066 (-0.116, -0.017) | 0.009 | -0.071 (-0.102, -0.040) | <0.001 | -0.002 (-0.027, 0.022) | 0.855 |
| **Model 6** | -0.069 (-0.119, -0.019) | 0.007 | -0.065 (-0.096, -0.034) | <0.001 | -0.012 (-0.037, 0.012) | 0.326 |
| **Model 7** | -0.054 (-0.107, -0.002) | 0.042 | -0.061 (-0.094, -0.029) | <0.001 | -0.008 (-0.034, 0.018) | 0.535 |

Note: All models were adjusted for age, body-mass index, skeletal muscle index at the first lumbar vertebra, and chronic obstructive pulmonary disease associated features: Model 1, visual emphysema grade (VEG) only; Model 2, total lung capacity (TLC) only; Model 3, square root of the wall area at internal perimeter of 10 mm (SRWA-PI10) only; Model 4, VEG and TLC; Model 5, VEG and SRWA-PI10; Model 6, TLC and SRWA-PI10; Model 7, VEG, TLC, and SRWA-PI10.

Note: Presented values were coefficients of pack-year with 95^th^ percentile confidence interval in parenthesis.

Abbreviations: C/L ratio, thoracic skeletal muscle index at the carina to skeletal muscle index at the first lumbar vertebra (L1-SMI) ratio; A/L ratio, pectoralis muscle index at the aortic arch to L1-SMI ratio; T/L ratio, erector spinae muscle index at the 12^th^ thoracic vertebra to L1-SMI ratio.

**Supplementary Table 3.** Detailed results of multiple linear regression analysis for skeletal muscle quality in the full model.

| **Variables** | **C-TSNAMA%** | ***P*-value** | **AA-PNAMA%** | ***P*-value** | **T12-ESNAMA%** | ***P*-value** | **L1-SNAMA%** | ***P*-value** |
| --- | --- | --- | --- | --- | --- | --- | --- | --- |
| **Pack-year** | -0.063  (-0.101, -0.025) | 0.001 | -0.076  (-0.141, -0.011) | 0.021 | -0.012  (-0.064, 0.040) | 0.643 | -0.040  (-0.076, -0.005) | 0.027 |
| **Age** | -0.309  (-0.434, -0.184) | <0.001 | -0.163  (-0.378, 0.053) | 0.138 | -0.361  (-0.534, -0.189) | <0.001 | -0.276  (-0.394, -0.158) | <0.001 |
| **BMI** | -1.468  (-1.765, -1.171) | <0.001 | -1.701  (-2.213, -1.189) | <0.001 | -1.560  (-1.971, -1.149) | <0.001 | -1.756  (-2.037, -1.476) | <0.001 |
| **L1-SMI** | 0.355  (0.208, 0.502) | <0.001 | 0.315  (0.061, 0.569) | 0.015 | 0.547  (0.343, 0.751) | <0.001 | 0.652  (0.513, 0.792) | <0.001 |
| **VEG (trace)** | 2.609  (0.229, 4.989) | 0.032 | 1.454  (-2.651, 5.559) | 0.486 | 0.323  (-2.971, 3.616) | 0.847 | 0.998  (-1.251, 3.248) | 0.383 |
| **VEG (mild)** | -0.290  (-2.605, 2.026) | 0.806 | 2.992  (-1.002, 6.987) | 0.141 | 0.436  (-2.768, 3.641) | 0.789 | 0.793  (-1.395, 2.982) | 0.476 |
| **VEG (moderate)** | -1.404  (-4.277, 1.469) | 0.337 | -2.970  (-1.002, 6.987) | 0.239 | -2.024  (-6.000, 1.951) | 0.317 | -2.899  (-5.614, -0.184) | 0.036 |
| **VEG (severe)** | -4.952  (-8.508, -1.396) | 0.007 | -4.118  (-10.251, 2.015) | 0.187 | -9.585  (-14.506, -4.664) | <0.001 | -4.816  (-8.177, -1.456) | 0.005 |
| **TLC** | -0.686  (-1.545, 0.173) | 0.117 | 0.145  (-1.336, 1.626) | 0.848 | -1.462  (-2.650, -0.273) | 0.016 | -0.116  (-0.928, 0.695) | 0.778 |
| **SRWA-PI10** | 0.259  (-3.470, 3.988) | 0.893 | -0.662  (-7.095, 5.771) | 0.840 | 0.018  (-5.143, 5.179) | 0.994 | 1.390  (-2.135, 4.915) | 0.438 |

Note: Presented values were coefficients of pack-year with 95th percentile confidence interval in parenthesis.

Abbreviations: C-TSNAMA%, percentage of normal attenuation thoracic skeletal muscle area at the carina; AA-PNAMA%, normal attenuation pectoralis muscle area at the aortic arch; T12-ESNAMA%, percentage of normal attenuation erector spinae muscle area at the 12^th^ thoracic vertebra; L1-SNAMA%, percentage of normal attenuation skeletal muscle area at the first lumbar vertebra; BMI, body-mass index; VEG, visual emphysema grade; TLC, total lung capacity; SRWA-PI10, square root of the wall area at internal perimeter of 10 mm.

**Supplementary Figures**

**Supplementary Figure 1.** Distributions of the percentage of a) low attenuation area less than -950 HU (LAA%950) and b) HU at 15^th^ percentile (HU15%) according to visual emphysema grade (0 – none, 1 – trace, 2 – mild, 3 – moderate, 4 – severe)


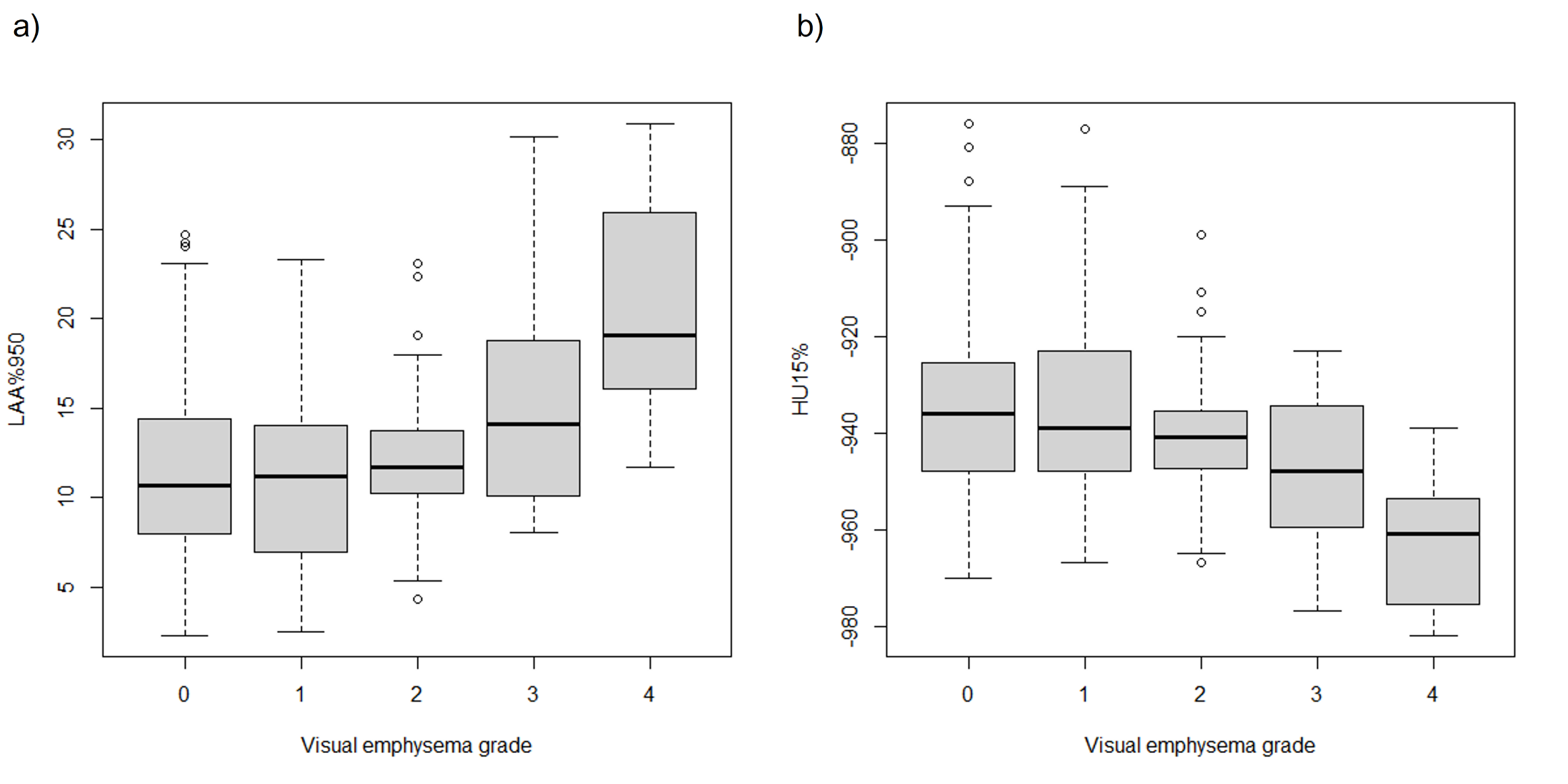


**Supplementary Figure 2.** Variable importance scores: a) thoracic skeletal muscle index at the carina (C-TSMI), b) percentage of normal attenuation thoracic skeletal muscle area at the carina (C-TSNAMA%), c) pectoralis muscle index at the aortic arch (AA-PMI), d) normal attenuation pectoralis muscle area at the aortic arch (AA-PNAMA%), e) erector spinae muscle index at the 12^th^ thoracic vertebra (T12-ESMI), and f) percentage of normal attenuation erector spinae muscle area at the 12^th^ thoracic vertebra (T12-ESNAMA%).


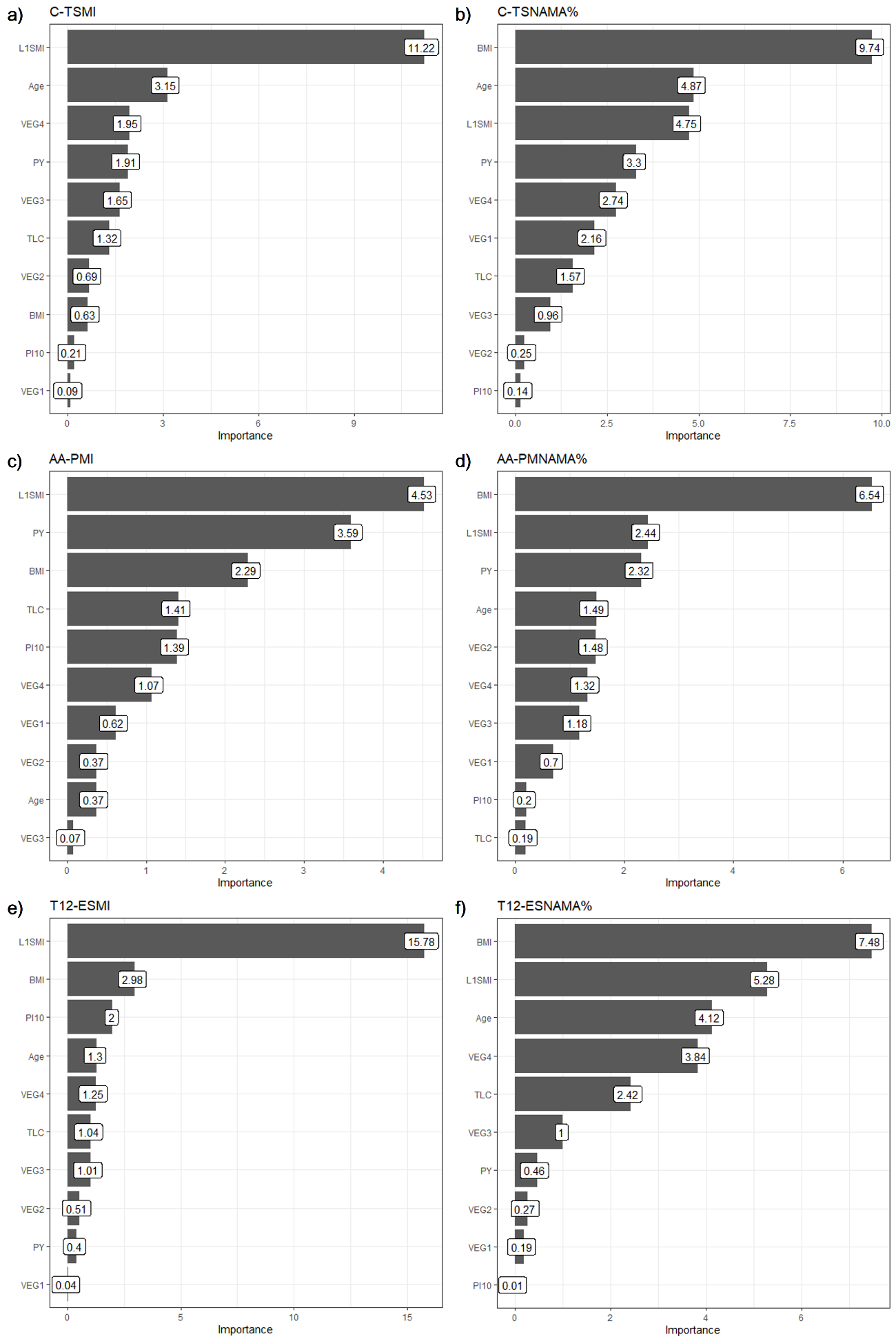


Abbreviations: L1-SMI, skeletal muscle index at L1 level; VEG, visual emphysema grade (1 – trace, 2 – mild, 3 – moderate, 4 – severe); PY, pack-year; TLC, total lung capacity; BMI, body-mass index; PI10, square root of the wall area at internal perimeter 10.
